# Refractory Orthostatic Hypotension Caused by a Recurrent Hemangioblastoma: Case Report and Review of the Literature

**DOI:** 10.7759/cureus.540

**Published:** 2016-03-24

**Authors:** Raj Nangunoori, Sravanthi Koduri, Anil Singh, Khaled Aziz

**Affiliations:** 1 Department of Neurosurgery, Allegheny General Hospital; 2 Drexel University College of Medicine; 3 Department of Pulmonary and Critical Care Medicine, Allegheny General Hospital

**Keywords:** orthostatic hypotension, posterior fossa tumor, hemangioblastoma, cervicomedullary junction, suboccipital craniotomy, von hippel lindau

## Abstract

Refractory orthostatic hypotension (OH) has been described following surgery for posterior fossa tumors. We present the case of a patient with refractory OH following attempted surgical resection. We also reviewed the available literature to describe pathophysiologic mechanisms for this rare entity.

A 58-year-old female was found to have a hemangioblastoma at the cervicomedullary junction following workup for dysphagia and coordination difficulties. She underwent successful suboccipital craniotomy and gross total resection. However, the patient’s symptoms returned several years later and a magnetic resonance imaging (MRI) showed tumor recurrence. A surgical resection was attempted but could not be performed due to significant scarring. Following discharge, she returned to our care with severe syncopal episodes, refractory OH, and an inability to ambulate. Aggressive medical therapy resulted in a gradual improvement in her ability to ambulate and a reduction in her orthostatic episodes. Unfortunately she died due to sepsis from aspiration pneumonia several months later. A survey of the literature yielded a total of 10 reports (14 patients) with refractory OH as a result of tumors in the cervicomedullary region. Five of fourteen patients died from complications related to OH and brainstem compression while the remainder had some improvement and were discharged.

Refractory OH can rarely be a presenting sign of a tumor in the cervicomedullary junction or can manifest following surgical resection of tumors in this region. Recognition of OH and the institution of medical therapy (sodium and fluid replacement) and pharmacotherapy may curb the significant morbidity associated with this condition.

## Introduction

Orthostatic hypotension (OH) is common among elderly patients and those with neurodegenerative disorders including the Shy-Drager syndrome, multiple system atrophy, Parkinson’s disease, diabetes as well as cerebrovascular disease [1-4]. Untreated, OH can severely limit functional independence in these patients. In the last several decades, there have been isolated reports of OH as a presenting sign of a new or recurrent posterior fossa tumor. We describe our experience with a 58-year-old female with refractory orthostatic hypotension following a recurrent hemangioblastoma with subsequent recovery following aggressive medical treatment with radiographic regression of her tumor. We obtained informed consent from the patient for this study. In addition, we surveyed the literature of prior reports to describe the pathophysiology that may cause the clinical symptoms associated with this rare phenomenon. 

## Case presentation

**Figure 1:**
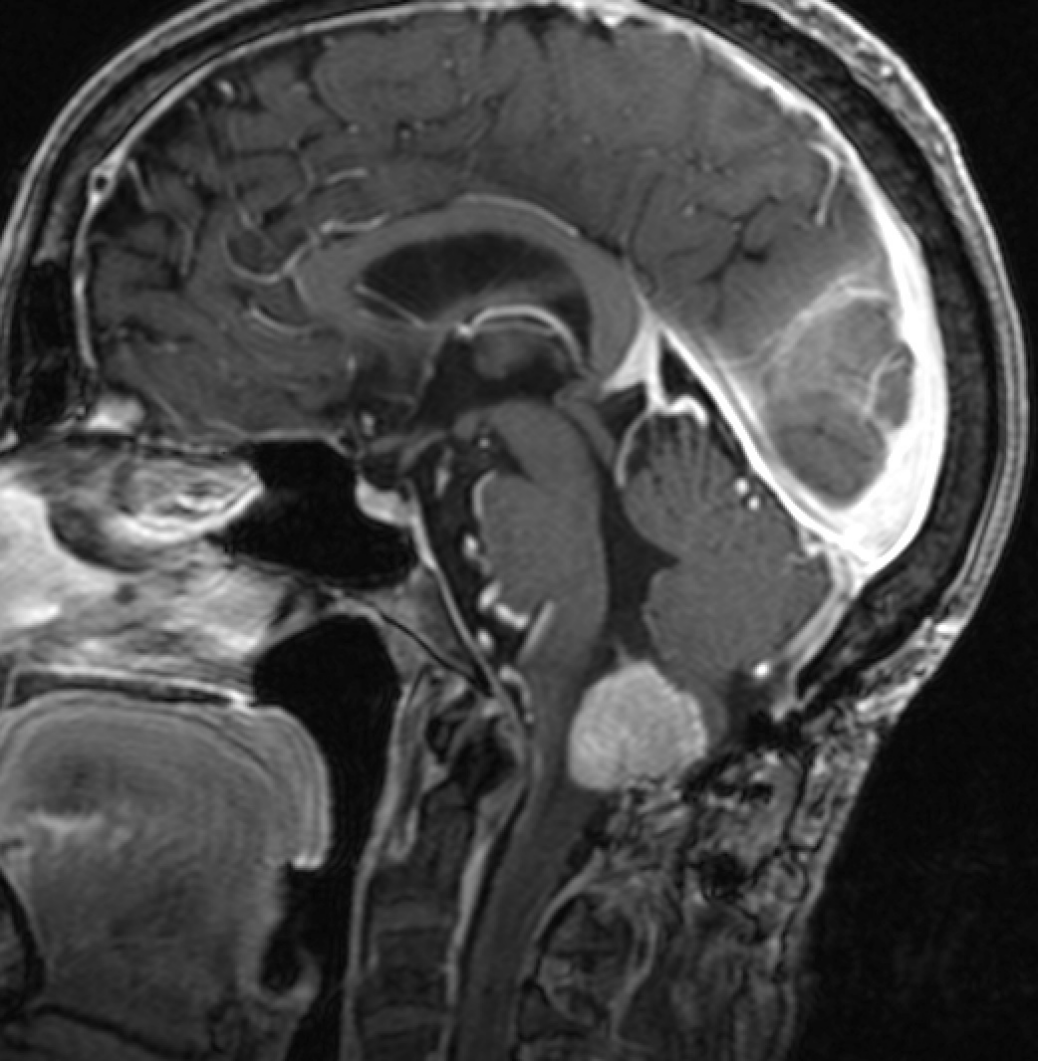
Recurrent Hemangioblastoma After Initial Resection MRI of the brain with contrast revealing a hemangioblastoma at the cervicomedullary junction.

A 58-year-old female first presented to our service in 2002 with chief complaints of “constriction” on the right-hand side of her body, dysphagia, coordination difficulties, and nighttime hiccups. She subsequently underwent a magnetic resonance imaging (MRI) of the brain revealing a 2 x 2 cm midline posterior fossa cyst with an enhancing mural nodule. The patient underwent suboccipital craniotomy and resection of the tumor that proved to be a hemangioblastoma. While her follow-up imaging initially showed no recurrence, the patient began to experience a return of her symptoms six years after surgery and underwent an MRI showing tumor recurrence (Figure 1) at the cervicomedullary junction.

Her neurologic examination revealed loss of her gag reflex and she underwent an attempted surgical resection in September 2013. Significant scarring and loss of landmarks prevented adequate surgical resection, so a biopsy of the tumor was taken and her prior suboccipital craniotomy was enlarged for decompression. The biopsy specimen confirmed recurrent hemangioblastoma. Postoperatively, the patient developed worsening dysphagia, ataxia of her upper extremities and aspiration pneumonia, necessitating gastrostomy tube placement. She was eventually discharged to inpatient rehabilitation on a low dose of maintenance dexamethasone (4 mg daily) for the significant edema caused by the tumor.

While undergoing rehabilitation, she was noted to have severe orthostatic hypotension resulting in fainting spells almost immediately upon attempting to stand or even sit upright. She was treated for her orthostatic episodes with an abdominal binder, fludrocortisone, and midodrine; however her fainting spells persisted. In one of her spells, she had jerking of her extremities that was interpreted as a seizure, and she was started on levetiracetam. Her severe orthostatic hypotension and fainting spells prompted transfer back to Allegheny General Hospital (AGH).

Upon her arrival to AGH, her orthostatic episodes were debilitating, preventing standing or sitting for more than 30 seconds without triggering a syncopal event. Multiple medical services, including Cardiology, Pulmonary/Critical Care, Endocrinology, Neurology, and Electrophysiology were consulted. Intravenous fluids were administered and her adrenal function was measured, revealing appropriate adrenal suppression given her chronic dexamethasone use. We began the institution of orthostatic vitals every eight hours on hospital day 3, and continued to treat her with midodrine (5 mg daily) and fludrocortisone (0.2 mg twice daily). Initially, the patient felt some subjective improvement in her orthostatic symptoms but continued to have syncopal episodes. On hospital day 5, with liberalized fluid intake and the aforementioned medications, her blood pressure ranged from 149-150 mmHg systolic and 87-106 mmHg diastolic without syncopal episodes. Because of her improvement with the use of midodrine, the frequency was increased to three times daily with a decrease in her fludrocortisone dose. In the following days, her symptoms recurred, with multiple syncopal episodes each time an attempt was made to stand.

**Figure 2:**
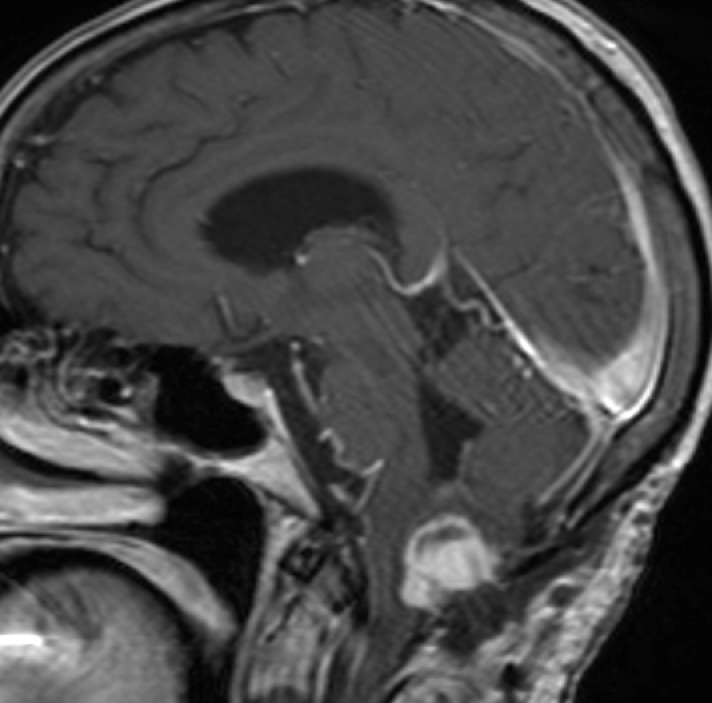
Hemangioblastoma After Attempted Surgical Resection Repeat MRI of the brain after attempted surgical resection showing no significant change in the size of the tumor or associated brainstem compression.

A repeat MRI of the brain (Figure 2) was performed showing no significant interval change in the size of the tumor or associated compression of the medulla. While able to mount an appropriate cardiac response, the patient continued to be orthostatic in spite of high dose IV dexamethasone (4 mg every six hours), midodrine (5 mg three times daily), fludrocortisone (0.2 mg twice daily), and IV fluids. Her midodrine was further increased to (10 mg three times daily) in response to her continuing orthostatic symptoms and she was mobilized within an hour of receiving her midodrine dose to allow for maximal benefit. In spite of being on fludrocortisone and maintenance fluids, she was found to have high urine output but was able to maintain fluid balance. Salt tabs and 3% hypertonic saline were instituted, as well as pyridostigmine. With these additions, the patient’s blood pressure ranged from 180-199/81-90 mmHg supine and 98-110/62-70 mmHg standing with fewer episodes of syncope and the ability to stand for several minutes at a time. Intolerable abdominal cramping led to discontinuation of the pyridostigmine several days later. She continued to have syncopal episodes, prompting the addition of albumin intravenously as well as DDAVP (Vasopressin) in an effort to improve her volume status and make her fluid balance positive. On hospital day 14, a Swan-Ganz catheter was placed to measure central venous pressure (CVP) and it was found to be 0; she was given additional volume and albumin. By hospital day 16, her orthostatic episodes were much less pronounced, with a supine blood pressure of 158/77 mmHg and standing blood pressure 123/108 mmHg and no syncopal events. Hypertonic saline was stopped on hospital day 19, and physical therapy was continued enabling her to stand for several minutes at a time. The patient was ultimately discharged to rehab on hospital day 21 on DDAVP (0.1 mg three times daily), salt tabs (2 g four times daily), IVF (normal saline at 100 ml/hr), midodrine (10 mg three times daily), and fludrocortisone (0.1 mg twice daily).

## Discussion

Orthostatic hypotension is defined as a fall in systolic blood pressure of at least 20 mm Hg or 10 mm Hg in diastolic blood pressure within three minutes of standing [3]. Symptoms of orthostatic hypotension include non-specific symptoms such as lightheadedness, syncope, neck and shoulder pain, and buckling of the legs [1-2]. Neurogenic orthostatic hypotension can be seen in a number of disorders, such as the Shy-Drager syndrome, pure autonomic failure, Parkinson’s disease, diabetes, amyloidosis, and in scattered reports, as the presentation of posterior fossa tumors, with or without surgical manipulation [1-14].

Central to the pathophysiology of orthostatic hypotension is a failure of the baroreceptor reflex, which involves a complex interplay of neural circuits. In a physiologically intact circuit, baroreceptor unloading from the carotid sinus and aortic arch decreases input to the nucleus of the solitary tract (NTS) in the dorsal medulla, decreasing vagal outflow and hence parasympathetic tone. The NTS, through excitatory projections to the caudal ventrolateral medulla, inhibits the rostral ventrolateral medulla, which acts on preganglionic thoracic spinal cord neurons. This disinhibition of the sympathetic preganglionic neurons allows for a vasoconstrictive response in response to a drop in systemic blood pressure. If blood pressure is not augmented by this response, norepinephrine/NE-containing neurons of the ventrolateral medulla, which project to the supraoptic and paraventricular nuclei of the hypothalamus, can activate vasopressin release [1-2].

Failure of the baroreceptor reflex arc can be through either the afferent or the efferent limb of the response. In many of these aforementioned reports (Table 1), the loss of the efferent limb of the baroreceptor reflex arc seems to be the underlying pathophysiologic mechanism behind the orthostatic hypotension seen in patients with posterior fossa tumors [3-14]. Since 1960, there have been at least 10 reports of this phenomenon, with a total of 14 patients. There has been a slight male preponderance (8:6) reported in the literature, with varying pathologic entities responsible, including ependymoma, meningioma, hemangioblastoma, cavernous angioma, and epidermoid tumors. Outcomes have been poor, with the death of 5/14 reported cases. In addition, the response to therapy, with both pharmacologic (fludrocortisone, midodrine, direct and indirect sympathomimetics) and non-pharmacologic interventions (abdominal binder, elastic stockings, conditioning exercises) have been mixed. The literature survey implies that this condition carries with it significant morbidity due to direct compressive effects on the medullary centers previously described as crucial for the maintenance of systemic blood pressure.

**Table 1:** Case Reports of Orthostatic Hypotension Related to Posterior Fossa Tumors Abbreviations: WBRT = Whole Brain Radiation Therapy, F = female, M = male, VPS = Ventriculoperitoneal shunt, HTN = hypertension ^§^Cardiac Pacer ^¥^As no surgical resection performed, imaging used to make presumptive diagnosis ^€^Able to ambulate six months after initiation of midodrine ^φ^Improvement *after* VPS placed, no orthostatic episodes ^ξ^Orthostatic blood pressure with tilt table test, but able to maintain erect posture for 30 minutes

Table 1. Case Reports of Orthostatic Hypotension Related to Posterior Fossa Tumors					
Author (Year)	Age, Gender	Pathology	Surgery?	Medical Treatment	Outcome
Riedel 1973	1: 50/M 2: 58/F	1: Ependymoma 2: Meningioma	1: Yes x 2 2: Yes	1: Postural conditioning, abdominal binder, compression stockings 2: Postural conditioning, compression stockings, fludrocortisone, salt tabs	1: Discharged to rehab 2: Ambulatory with quad cane
O’Malley 1979	1: 40/F 2: 32/F	1: Hemangioblastoma 2: Histoplasma granuloma	1: No 2: Yes	1: Oral ephedrine: ineffective 2: None	1: Death 2: Resolution of symptoms
Telerman 1982	55/M	Unknown	No	Fludrocortisone 3x daily helped with ataxic symptoms	Death
Hsu 1984	1: 16/M 2: 37/M 3: 69/M	1: Anaplastic astrocytoma 2: Hemangioblastoma 3: Metastatic oat cell carcinoma	1: Yes + radiation 2: Yes + radiation 3: Yes^§^	1: Phenylephrine, atropine, propranolol 2: Salt loading/physical therapy (ineffective), fludrocortisone (effective) 3: Salt/fluid loading, fludrocortisone, ephedrine, body binder, physical therapy	1: Death 2: Death 3: Death
Yamashita 1994	54/M	Possible Lymphoma^¥^	No; + WBRT	Sympathomimetic/mineralocorticoid agents ineffective; compression stockings	Ambulate w/o assistance occasionally
Jabary 2007	54/F	Hemangioblastoma	Yes + VPS	Fludrocortisone, caffeine, ergotamine, ephedrine (ineffective); abdominal binder, postural conditioning, compression stockings, midodrine (effective)^€^	Ambulatory 5 years post-op, no tumor recurrence
Tadros 2009	54/M	Subependymoma	Yes x 2	Fludrocortisone, isometric hand grip exercises	
Gomez 2009	48/M	Epidermoid	Yes + VPS	Fludrocortisone, midodrine (no benefit), compression stockings	Improvement in OH^φ^
Idiaquez 2009	51/F	Cavernous angioma	Yes	Fludrocortisone, midodrine	Improvement in OH^ξ^
Hocker 2012	34/F	Hemangioblastoma	Yes	Labetalol, liberalization of oral salt intake (gatorade, etc), salt tabs, midodrine, lisinopril (prevent supine HTN)	Ambulate w/o assistance

Treatment of OH and its associated symptoms include adequate hydration, conditioning exercises to prevent venous pooling, avoidance of anti-hypertensive medications, and the use of medications. In our patient, she had already been fitted with thigh-high stockings and started on midodrine for treatment. Our patient’s pharmacotherapy was initially reduced to allow for a better assessment of the cause of her OH. Her echocardiogram revealed no abnormalities, and as expected, the patient appropriately mounted a tachycardia upon changing position from supine to standing. Despite her tachycardia, her blood pressure remained low, suggesting that the efferent limb of the baroreceptor arc was affected by medullary compression and she was unable to increase her peripheral vascular resistance. Adrenal insufficiency was ruled out as the patient had been on long-term dexamethasone since her attempted surgical resection. A Swan-Ganz catheter placement revealed that the central venous pressure (CVP) was 0, suggesting hypovolemia in spite of being on intravenous fluids, DDAVP, midodrine, salt tabs, and fludrocortisone.

**Figure 3:**
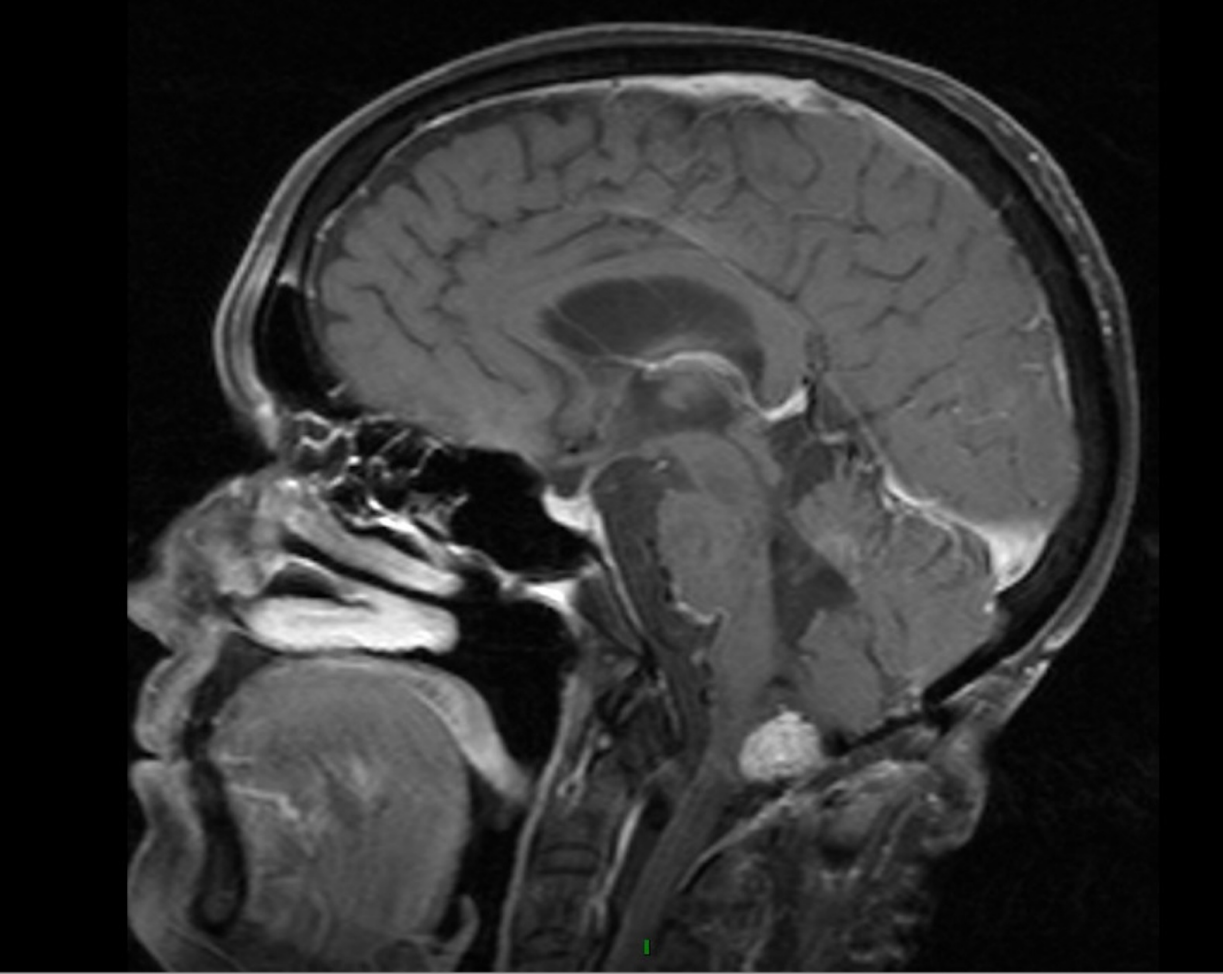
Regression of Tumor MRI of the brain showing decrease in size of patient’s hemangioblastoma at last follow-up.

In our particular case, the patient had an uneventful surgical resection initially and presented with a recurrence almost 11 years later. While gross total resection could not be performed, it is possible that surgical manipulation alone may have contributed to the patient’s symptoms. Unlike previously reported cases, our MRI at the time of attempted resection compared with the time of the patient’s severe orthostatic episodes did not show any change in the amount of edema or degree of medullary compression. Additionally, in our patient, other etiologies of hypotension had been ruled out with diagnostic and laboratory testing. Her waxing and waning responses to medications in light of thorough medical evaluation for alternative causes of her OH implicate a central etiology. She returned to clinic six months after her hospitalization and had a near resolution of her symptoms, had the ability to ambulate short distances with a walker and a reversal of her gastrostomy tube. An MRI performed at her latest follow-up visit (Figure 3) showed regression of her tumor, further advancing our hypothesis that medullary compression by her known hemangioblastoma was causative in producing her severe refractory OH. Unfortunately, in spite of her tumor regression, the patient succumbed to pneumonia secondary to aspiration and went into acute respiratory distress syndrome (ARDS) and passed away at an outside institution.

While there are scattered reports of posterior fossa tumors causing this phenomenon, including hemangioblastoma [6,8,10, 14], we present the first case of a patient with truly refractory OH in spite of maximal medical therapy, in whom surgical resection was not possible and neuroimaging revealed no progression in the size or associated medullary compression. Aggressive medical treatment with volume replacement and medications to augment her peripheral vascular resistance and intravascular volume were instrumental in restoring her ambulation.

## Conclusions

We believe that while extremely rare, patients with posterior fossa tumors, particularly at the cervicomedullary junction should be counseled regarding development of severe OH following surgery. In addition, prudent observation of patients following surgery for tumors in this location is important as is the institution of aggressive medical therapy to decrease the morbidity of this condition following surgery in this region. 
